# Detecting Alu insertions from high-throughput sequencing data

**DOI:** 10.1093/nar/gkt612

**Published:** 2013-08-05

**Authors:** Matei David, Harun Mustafa, Michael Brudno

**Affiliations:** ^1^Department of Computer Science, University of Toronto, 10 King's College Road, Toronto, ON M5S 3G4, Canada and ^2^Centre for Computational Medicine, Genetics and Genome Biology Program, The Hospital for Sick Children, 555 University Avenue, Toronto, ON M5G 1X8, Canada

## Abstract

High-throughput sequencing technologies have allowed for the cataloguing of variation in personal human genomes. In this manuscript, we present alu-detect, a tool that combines read-pair and split-read information to detect novel Alus and their precise breakpoints directly from either whole-genome or whole-exome sequencing data while also identifying insertions directly in the vicinity of existing Alus. To set the parameters of our method, we use simulation of a faux reference, which allows us to compute the precision and recall of various parameter settings using real sequencing data. Applying our method to 100 bp paired Illumina data from seven individuals, including two trios, we detected on average 1519 novel Alus per sample. Based on the faux-reference simulation, we estimate that our method has 97% precision and 85% recall. We identify 808 novel Alus not previously described in other studies. We also demonstrate the use of alu-detect to study the local sequence and global location preferences for novel Alu insertions.

## INTRODUCTION

### Background

With high-throughput sequencing (HTS) becoming a standard methodology for the characterization of human genomes, it has become essential that we have effective methods to identify all types of variants from HTS data. Although there has been extensive work in designing methods that can identify both small-scale SNPs and indels [see reviews (1,2)] as well as large-scale copy-number variation (3,4), fewer methods have been developed that can effectively identify the variability in repeat content of human genomes directly from non-targeted HTS data (5–8). The most common repetitive element in the human genome is the Alu, a retrotransposon that is ∼300 bp in length, with 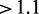 million copies, representing >10% of human DNA (9). The three families (AluJ, AluS and AluY) and ∼30 subfamilies of Alus have been active at different points during primate evolution (10–12), and they play important roles in genome modification by assisting in the creation of structural variants (13,14). Although only some subfamilies of AluY are still capable of retrotransposition (15), the identification of novel Alu insertions in personal genomes is of medical interest, as they have been linked to several disorders (16–22). For more information on Alus, see the excellent reviews (23) and (20).

### Alu repeats: replication and distribution

**Figure 1.**
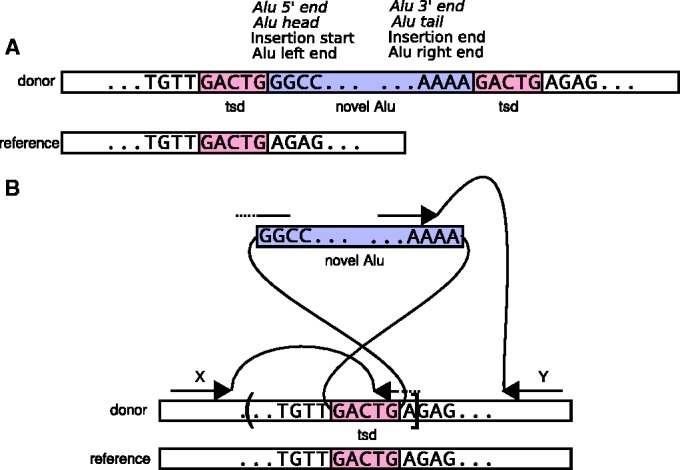
Detecting Alu insertions and TSDs. (**A**) Genome after an Alu insertion on the positive strand. The human reference genome is at the bottom. The newly sequenced ‘donor’ genome with an Alu insertion and a TSD is above. Above is the terminology. ‘Head’ and ‘tail’ refer to the inner sequence of the Alu, whereas ‘left’, ‘right’, ‘insertion start’ and ‘insertion end’ refer to the orientation of the genome. If the Alu were on the negative strand, the terms in italic would be flipped. (**B**) Detection of Alu insertions. The donor genome is shown aligned to the reference genome. Because of the TSD, the left end of an Alu will start at the right end of the TSD. Two read pairs supporting the insertion are shown. Read pair X has a split-mapped read aligned across the 5′ (left) end of the Alu (GGCC), and the right end of the TSD, directly identifying the breakpoint. Read pair Y supports the presence of an insertion, but does not identify the exact breakpoint (other end of the TSD). As only the left endpoint is detected (between G/A), the right end of the confidence interval is the A following the breakpoint, whereas the left is only estimated. The detected breakpoint is represented by a square bracket, and the undetected one by a round bracket.

The Alu is a Short Interspersed Element, which multiplies using a ‘copy and paste’ mechanism. In the ‘copy’ phase, Alus are transcribed by RNA polymerase III. For the ‘paste’ phase, Alus use a ribonucleoprotein complex composed of proteins encoded by a different retrotransposon, the Long Interspersed Element L1. L1 transcripts produce endonuclease (EN) and reverse-transcriptase (RT). EN initially cleaves one DNA strand, and RT copies an Alu transcript into a single strand of DNA at that location. The second DNA strand is cleaved by an unknown mechanism, and then the DNA repair mechanism generates the strand complimentary to the novel Alu insertion. The process is called Target-Primed Reverse Transcription (TPRT) (24–27). Because of the two distinct single-strand breaks, the final DNA sequence contains a Target Site Duplication (TSD), which is a sequence of 4–25bp repeated just before and just after the new Alu element (see Figure 1A).

The initial EN cleavage site shows a preference for certain sequences, notably the TT/AAAA motif (the notation emphasizes that the cut occurs between the T and the A) (26,28,29). Several manuscripts have studied both common sequences at the nick sites, as well as broader genomic context of Alu insertions, including their frequency in coding regions, or in the vicinity of other Alus.

In these studies, it is necessary to make a distinction between analysing *fixed* Alus, which occur in all humans, and *polymorphic* Alus, present only in a fraction of the population. Fixed and polymorphic Alus are subject to different types of pressure: while there are functional constraints preventing both novel polymorphisms and fixed Alus from appearing in regions such as exons, fixed Alus have been in the genome long enough to be involved in additional events and may be removed through unequal recombination (30). Similar to single nucleotide substitutions and other polymorphisms, only a small fraction of Alus in the human genome are polymorphic. Although the reference human genome has 1.1 million Alus, most studies identify <2000 novel Alus in an individual human genome (6–8), and the single largest polymorphism study by (8) found <8000 polymorphic Alus in 185 individuals of diverse ethnicities.

Many studies have used Alu annotations in the human reference genome to correlate the locations of (implicitly) fixed Alus with various other features of the genome: GC content (30), segmental duplications (31), other Alus (28), other Alus in intergenic and intronic regions (32,33) and other Alus in sense and antisense orientations (34,35).

Others have considered the distribution of polymorphic Alus in the genome. NCBI and Celera genomes were compared to study polymorphic Alus in (29). They found that Alu insertions are inversely correlated with genes and positively correlated with known integration sites. Novel Alus are significantly depleted in exons and introns (6). Novel Alus were used to quantify depletion in exons and coding exons (8).

Although most studies agree that Alu insertions are depleted in both coding and non-coding exons, there is some disagreement on whether they are also depleted in introns: depletion of polymorphic Alus in introns is observed in (6). Under the natural assumption that Alus are mainly subject to negative selection pressure, a depletion of polymorphic Alus should result in a depletion of fixed Alus as well. However, (33) finds that fixed Alus have similar density in introns and intergenic regions.

Furthermore, polymorphic Alus are most likely subject to different types of selection pressure in living organisms and in tissue culture systems. For instance, (36) considers the distribution of tagged Alu insertions in *ex vivo/in vitro* assays with minimal selection pressure. In contrast, we expect that most novel Alus we find were inserted under regular selection pressure *in vivo*.

### Detection of Alus from HTS data

In earliest studies, the detection of Alus was conducted with targeted methods such as PCR followed by one-by-one genotyping of loci of interest (37–39), most recent projects identify novel Alus in a genome-wide manner, either by comparison of assembled genomes (29,40) or, more recently, by direct analysis of unassembled reads generated by HTS technologies. In particular, (6) was the first to address this problem. They used the read-pair based VariationHunter algorithm (41), which computes the minimum number of clusters of paired reads where one end maps to an Alu and the other maps to the genome. They analysed eight Illumina samples, finding about 1144 novel Alus per individual. An alternative implementation of a read-pair-based algorithm was proposed by (42). They found an average of 5990 novel Alus per individual, an unusually large number compared with other studies (including ours).

A comprehensive map of mobile element insertion polymorphisms, including Alus, was presented by (8), based on the 1000 Genomes Project pilot 1 (low-coverage) and 2 (high-coverage trios) data sets of whole-genome sequencing (WGS) data from 185 samples (43). Their approach used both Illumina paired reads and longer unpaired Roche/454 data. They used the read-pair-based Spanner algorithm on the Illumina reads to identify variant locations and split mapping of the Roche/454 reads with the Mosaik algorithm to identify the breakpoints. This method provides breakpoint resolution, but only from Roche/454 data. Their analysis identified on average 921 novel Alus per high-coverage sample, and 490 novel Alus per low-coverage sample.

Most recently, (7) developed the Tea pipeline, which combines read-pair and split-read approaches to detect novel transposable elements, including Alus. They analysed three Yoruban samples with 40× average coverage and identified on average 1037 novel Alus per sample, as well as 41 germline samples from cancer patients with 35× average coverage with 742 novel Alus per sample. This was the first method to use Illumina data for Alu breakpoint identification.

### Our contribution

In this study, we present a new Alu detection method called alu-detect. Our method identifies Alu breakpoints and TSDs while also identifying insertions without exact breakpoint information. The alu-detect works with both WGS and exome-capture data and identifies Alu insertions in the vicinity of reference Alus. We also study the distribution of Alus relative to other genomic features, including genic regions and other Alus.

## MATERIALS AND METHODS

In this section, we first present the alu-detect tool. Subsequently, we discuss a novel method for setting parameters for alu-detect, based on a faux-reference simulation of Alus in the human genome. Finally, we describe the HTS data sets we used, as well as the Alu call sets made by other studies to which we compared our results.

### The Alu-detect tool

#### Overview

The major steps of the alu-detect algorithm are as follows:

1. Given the mappings of a HTS data set to the reference, identify reads or read pairs (henceforth called ‘fragments’) that have evidence of insertions: poorly mapped reads and tails of reads or discordant pairs.
2. Remap these fragments to the reference while allowing for some parts of each fragment not to map. This allows us to detect the reference positions for fragments that span insertion breakpoints.
3. Detect evidence of Alu insertions by mapping each fragment to the set of consensus Alu sequences.
4. Construct clusters of fragments along the reference genome that have evidence of Alu insertions. If the fragments consist of paired reads, the pairing information (orientation and relative position) is taken into account during this step.
5. Investigate each fragment in the cluster with a split-mapping algorithm, which allows the alignment to jump from the reference to an Alu consensus sequence and back (see Figure 1B). Each candidate novel Alu is detected with neither, one or both breakpoints.
6. Filter candidate insertion calls based on their support, including number of supporting fragments, length of the inserted Alu and quality of the read mapping. In this study, for every WGS data set, the filtering thresholds were set independently and in an automated way.

#### Discordant reads and initial mapping

The alu-detect tool takes as input a reference genome (usually the NCBI human genome) and the mappings of a donor read set to this reference (a SAM or BAM file), including unmapped reads. The program detects the read pairing information: whether the reads are paired and, if so, the minimum and maximum fragment sizes, discarding 0.1% outliers. In the process, it is assumed that pairing information is consistent for all reads from the same read group. Next, the reads with discordant mappings (either partial mappings, discordant insert sizes or paired reads where only one is mapped) are extracted for further investigation. These reads are progressively mapped in unpaired mode to the reference genome using Bowtie2 (44): if a read does not map, it is trimmed from the 5′ end until it maps or until it becomes <20 bp. This is then repeated by trimming from the 3′ end. For every read pair, if at least one read is mapped with a mapping quality of at least 5 to the reference, that pair is kept, and the rest are discarded. The remaining reads are mapped to the set of Alu consensus sequences using SHRiMP2 (45).

#### Split mapping

The next step is the core of the method. The remaining reads are now clustered based on their (or their mate’s) partial mapping to the reference, taking into account position, strand and pairing information. Clusters of reads with Alu evidence are then remapped to the reference using a split-mapping algorithm. Concretely, each read is aligned to the reference using the Smith–Waterman algorithm (46), and the alignment is allowed to jump between the reference and an Alu consensus sequence using the algorithm described by (47).

For every cluster of reads, the program computes: the mapping score of all reads assuming an Alu insertion, the mapping score for the null hypothesis (mapping all reads to the reference only) the read pair support for the Alu insertion, the minimum and maximum positions inside the Alu that were mapped and the location of one or both breakpoints.

#### Alu terminology and confidence intervals

The terminology we use to describe the location and orientation of Alus is illustrated in Figure 1. We say that an Alu is inserted ‘on the positive strand of the reference genome’ if its 5′ end appears left of its 3′ end on the positive strand of the genome. Otherwise, we say it is on the negative strand. We use the term ‘Alu head’ to refer to the 5′ end of an Alu, and the term ‘Alu tail’ to refer to the 3′ end of an Alu, including its poly-A tail. When using terms such as ‘left end’, ‘right end’, ‘insertion start’ and ‘insertion end’, we implicitly refer to the positive strand of the reference genome. Thus, for example, if an Alu is inserted on the positive strand of the reference genome with a regular TSD (see Figure 1B), the terms Alu head, Alu left end and insertion start all refer to the 5′ end of the Alu, which is connected in the donor to the right end of the left copy of the TSD. Likewise, the terms Alu tail, Alu right end and insertion end all refer to the Alu 3′ end and poly-A tail, which is connected in the donor to the left end of the right copy of the TSD.

**Figure 2.**
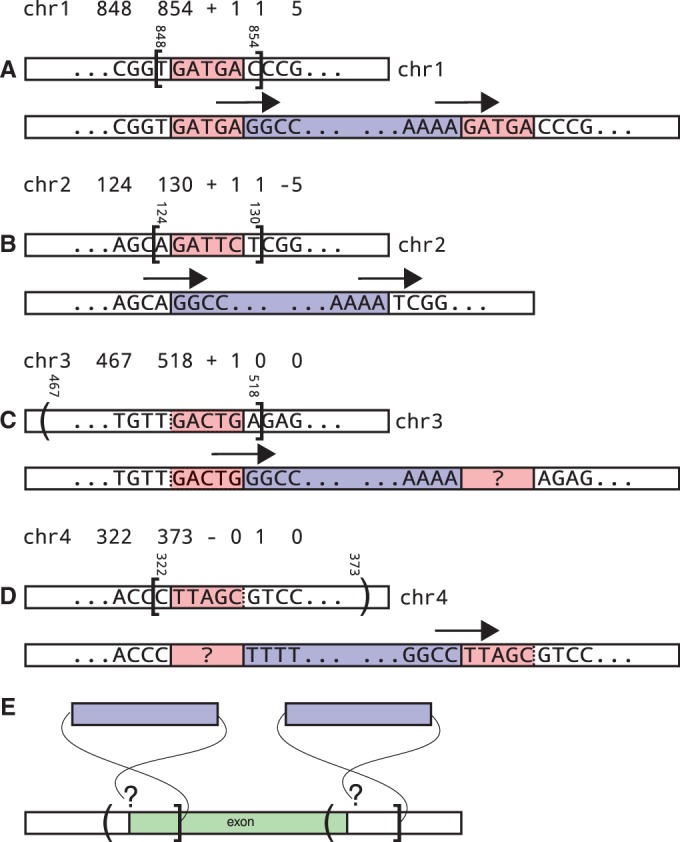
(**A–D**) Illustration of the Confidence Intervals. The text line shows the chromosome, the confidence interval start, end, the strand of the insertion, the number of reads spanning the left endpoint/start of the insertion, the number of reads spanning the right endpoint/end of the insertion and the reported TSD length. The diagrams show the reference on top and the inferred donor genome on bottom. Arrows denote the reads supporting the breakpoints. A bracket denotes a confidence interval end next to which a breakpoint was detected. A paranthesis denotes an end, which is only an estimation. (A) Standard call with two breakpoints and TSD. (B) Non-standard call with two breakpoints, showing a target site loss. (C) Call with only the left endpoint detected. Assuming the insertion has the standard form, the TSD starts somewhere upstream of the breakpoint in the reference (the uncertainty is represented by the dotted line). The region marked with the ‘?’ is the second copy of the TSD; its starting sequence is not known. (D) Similar to C, but insertion with only the right endpoint detected. (**E**) Alu Calls and Genome Features. The reference and a genomic feature (exon) are shown, together with the confidence intervals for two Alu insertion calls. Each Alu’s left breakpoint is detected, whereas the right is estimated. Only the left call is guaranteed to duplicate part of the genome feature. For the right call, this depends on the undetected right end of the insertion (TSD length).

Alu-detect reports a ‘confidence interval’ (CI) for each Alu call (we use the term ‘confidence interval’ to emphasize the location uncertainty and not a statistical confidence value.) (see Figure 2A–D). If neither breakpoint of the call is detected, the CI is computed based on the read pairs that support the call, including the pairing information. If both endpoints are detected, one between *x* and *x* + 1, the other between *y* and *y* + 1, the CI is 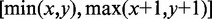.

To understand CIs when only one endpoint is detected, refer to Figure 2C and D. If only the left endpoint is detected between positions *x* and *x* + 1, the CI is 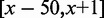. Similarly, if only the right endpoint is detected between positions *x* and *x* + 1, the CI is 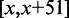. This convention ensures that for all ‘usual’ Alu insertions, where the TSD is almost never 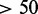 bp (27), the entire TSD is included in the CI. As a result of this convention, two independent runs, e.g. on related individuals, which detect different breakpoints of the same ‘usual’ Alu insertion will have overlapping CIs for that insertion.

When only one breakpoint is detected, the fact that the CI of an Alu call intersects a genomic feature is not a guarantee the Alu insertion would actually alter that feature. See Figure 2E. Concretely, say a genomic feature occurs at positions (1, 100), and the CI of an Alu call is (71, 130), detected with one breakpoint only. If the insertion start breakpoint is detected, that occurs between positions 129 and 130; therefore, the insertion intersects the genomic feature only if it has a TSD longer than 29 bp. On the other hand, if the insertion end breakpoint is detected, that occurs between positions 71 and 72; therefore, the insertion definitely interrupts the genomic feature.

#### Filtering

Novel Alus are intially detected by alu-detect based on only two supporting fragments (we refer to these as ‘unfiltered’ calls). To reduce false-discovery rate and produce high-confidence Alu calls, we use several support parameters to filter the detected insertions. The filters are based on confidence interval length (ci-len), span of inner Alu positions mapped (len), read pair support for the call (supp) and difference between Alu call score and read mapping null hypothesis score (null). The parameters for filtering are set automatically as explained in the next section.

Predicting Alu insertions in repetitive regions using HTS data is naturally limited by the mappability of the reads to the reference genome. To produce an Alu insertion call in a certain region, a necessary (but not sufficient) condition imposed by alu-detect is that at least one fragment with potential Alu evidence be mapped to that region with mapping quality five or more. Consequently, alu-detect cannot produce calls in regions where all mappings have quality 0 (e.g. perfectly duplicated regions in the reference). The alu-detect does not differ significantly form other tools in percentage of calls in repetitive regions, except for near reference Alus, which other methods often make no, or fewer calls.

In addition to the filters described earlier in the text, we also require Alu calls overlapping reference Alus to be supported by at least one non-ambiguous breakpoint. To explain this, assume the reference contains an Alu on the positive strand, between locations (101, 400). An Alu call on the positive strand with CI (90, 110) would pass this filter if and only if its right end (tail) breakpoint is detected. The rationale is that the left end (head) of the Alu call is close to the head of the reference Alu at 101, and deviations in the reference genome from the Alu consensus sequence might cause the donor reads to map better to the consensus sequence than to the reference Alu. The same does not apply for the right end (tail) of the Alu within the given CI: no similar sequence exists in the reference until position 400. Thus, detecting the right end (tail) is a more certain indicator the call is real.

### Automated parameter selection

Most methods for identifying genetic variants allow the user access to a number of parameters that, when properly set, maximize the performance of the method on the specific data set. Although such parameters add to the flexibility of the tool, they are often difficult to select, and performance can be degraded if the parameters are inappropriate. In the case of alu-detect, such parameters include required number of supporting fragments, length of the inserted Alu sequence and difference in alignment scores between aligning to the Alu and to the reference. One potential method to set the parameters would be simulation of a data set that is similar to the input one, but with a known ground truth. For detecting Alu insertions, a ‘standard’ simulation experiment would consist of the following:

- Adding Alu insertions to the human reference genome to create a faux donor
- Simulating HTS reads from this donor genome, typically using uniform distribution and some error model for the individual nucleotides

Then, one runs the detection method on the resulting data, using the information from step (i) as ground truth to compute the precision and recall and chooses the parameters that offer best performance trade-offs. In practice, such an approach is not used because both steps (i) and (ii) are independently unrealistic: the Alus added in step (i) might not follow the natural distribution of novel Alus found in a real human genome and also are not going to reflect the continuing evolution of the Alu repeats. Simultaneously, the HTS reads produced by the simulation in step (ii) are unlikely to follow the distribution of reads from a real sequencing experiment.

To set the parameters of alu-detect, we use alternate, faux-reference simulation experiments. We first contruct a ‘fake’ reference genome by removing from the real reference genome all sequences, which resemble novel Alus: AluYs of at least 310 bp in length, and immediately surrounded by a perfect TSD of at least 6 bp. These criteria were motivated by the idea to select reference Alus that were inserted by TPRT (hence the TSD requirements) and looked similar to novel Alus (young families, with large length). In all, 10 440 reference Alus were selected. We created a new ‘fake’ reference by removing all of these Alus, along with one copy of the TSD, thus computationally reversing TPRT mechanism.

We then provide this faux reference genome to alu-detect, together with a real HTS data set to generate a set of Alu insertion calls. Each of these calls is either a simulated variant or a true polymorphism (differences between the original reference and the sequenced donor). To reduce the impact of such polymorphisms on the results, we filter the set of calls for all known Alu polymorphisms from the data sets of (6,7,8,48). The remaining Alu calls were our positives (P). Every Alu call that intersected the remaining TSD associated with a removed Alu was labelled a true positive (TP), the rest were labelled false positives (FP). ‘Simulated’ precision and recall were then defined as usual: 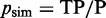 and 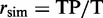 (where T = 10 440 was the number of Alus removed).

We then took Alu calls made on the faux reference and filtered them in multiple ways (see previous section for filter definitions): len from 150–290 in steps of 10; supp 6–25; null 0–30% in steps of 5% and ci-len 300–1100 in steps of 200. For every precision threshold *x*, we compute the filter parameters that maximize 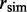, subject to 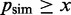. These parameters are then used to run alu-detect with the real reference and the same HTS data set.

#### Evaluation of automated parameter selection

**Figure 3.**
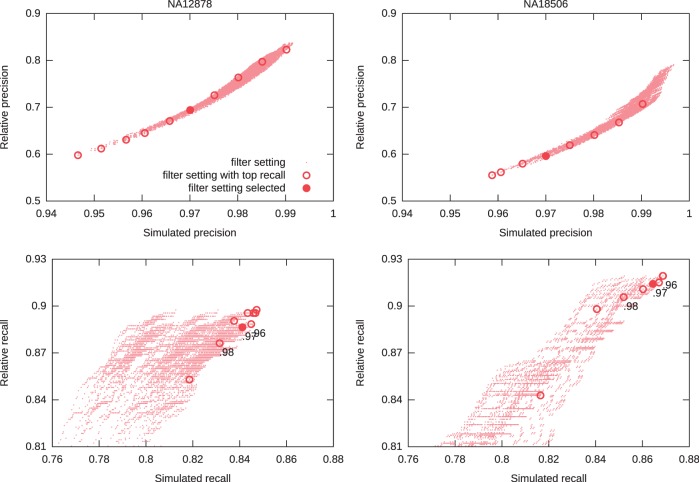
Top: Relative Precision (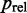) versus Simulated Precision (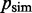). Bottom: Relative Recall (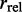) versus Simulated Recall (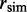). Each dot represents a filter setting. We highlight, for *x* between 0.920 and 0.990 in steps of 0.005, the filter with 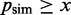 and maximum simulated recall 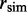. NA12878 is an individual of European ancestry, whereas NA18506 is an individual of Yoruban ancestry.

To show that our simulated precision closely correlates with performance at detecting known Alus, we computed the ‘relative’ precision 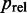 as the percentage of Alu calls made (using the real reference) that are present in either (8) or (48). Figure 3 (top) shows the close correlation between 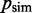 and 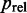 on the HTS data sets for NA12878 (CEU individual) and NA18506 (Yoruban) for all thresholds under consideration. 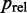 will vary widely between individuals, as different individuals have a different fraction of novel Alus already characterized in the two data sets.

To have a single set of results to compare our method to other studies, we have chosen a simulated precision cutoff of 0.97 that produces a relative precision values 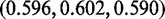 on the Yoruban trio (NA18506, NA18507, NA18508), comparable with 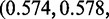 0.576) achieved by (6). Thus, every HTS data set was filtered with settings corresponding to maximum 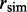, given that 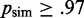.

We present a similar plot in Figure 3 (bottom), showing the connection between simulated recall 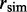 and relative recall 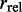. Relative recall is harder to estimate than relative precision because we need a set of known Alu insertion calls for the specific individual in question. For this plot, we used the calls made by (8) on NA12878 and the calls made by (7) on NA18506 as relative truth for those two individuals. For values of simulated precision greater than ∼0.97, the correlation between relative and simulated recall becomes weaker because of the true novel insertions in the donor (i.e. polymorphisms).

### Alu insertions near reference Alus

To analyse the distribution of Alu insertions near reference Alus, we selected those reference Alus that are at least 200 bp away from other Short Interspersed Elements, whose annotation starts before position 10 (of the corresponding consensus sequence) and ends between positions 290 and 350, and where the difference between the Alu lengths in the genome and in the consensus sequence is not >10%. The reason for these restrictions was to have clear non-conflicting information for the start and end regions of each reference Alu under consideration. Notably, because of these restrictions we do not consider insertions near clusters of reference Alus. In total, we selected 443 292 of 1.1 million reference Alus for our analysis. We refer to these as ‘clear’ Alus.

**Figure 4.**
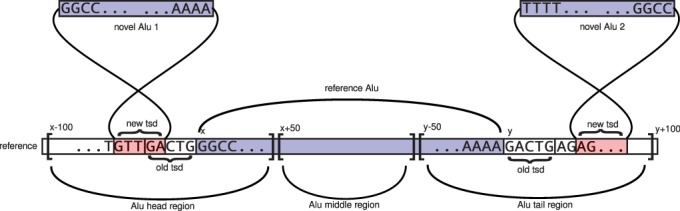
Alu insertions next to a reference Alu. We show the relevant regions around a reference Alu, along with two novel Alu insertions with their reference mappings. Novel Alu one is inserted on the positive strand of the head region of the reference Alu, and novel Alu two is inserted on the negative strand of the tail region of the reference Alu.

We analysed insertions in three distinct regions related to reference Alus: the ‘Alu head region’, starting 100 bp upstream of the 5′ end of a reference Alu and ending 50 bp downstream from it [as observed by (19), this region contains the original site where EN cleaved the genome during the insertion of this Alu]; the ‘Alu middle region’, starting 50 bp downstream from the 5′ end, and ending 50 bp upstream from its 3′ end (this region contains the A-rich linker between the two dimers that form an Alu); and the ‘Alu tail region’, starting 50 bp upstream from the 3′ end, and ending 100 bp downstream from it (this includes the poly-A tail of the Alu). We say that an Alu is inserted ‘on the positive strand of a reference Alu’ if it is inserted in the same orientation as the reference Alu. We say that an Alu is inserted ‘on the positive strand in the head region of a reference Alu’ if the Alu is inserted in the same orientation as the reference Alu, and the TSD of the novel Alu intersects the head region of the reference Alu. For reference, see Figure 4.

### Preparation of other Alu call sets

The Alu calls in dbRIP (48) typically include both breakpoints of the insertions, along with the flanking region. The database includes both novel Alus and reference Alus; however, we use only the novel ones. Each of these calls was enclosed in a confidence interval of length equal to its TSD (as included in the dbRIP record).

Each Alu call from (6) includes a confidence interval. In our analysis, we expanded the confidence interval to a minimum of 20 bp to include a potential TSD.

The Alu calls of (8) include both insertions and deletions. We only use the insertion data. Each Alu insertion includes a confidence interval and possibly a TSD. In our analysis, we added the TSD to the confidence interval.

A final source of Alu calls was (7). All calls in these data are reported with both breakpoints, and the confidence interval includes the TSD. We disregarded Alu insertions without strand information.

Comparing 3 (or more) sets of Alu calls (intervals) is non-trivial because interval intersection is not transitive. In our evaluations, we used the multi-inter program that is part of the bedtools (49) suite (using the option -cluster).

### HTS data sets analysed

We analysed WGS data for seven individuals: one Yoruban trio NA18506 (ERX009608), NA18507 (ERX009609), NA18508 (ERX009610); one Central European (CEU) trio NA12877 (ERX069504), NA12878 (ERX069505), NA12882 (ERX069506); and one additional individual of unreported ancestry SRS228129 (SRX083311, SRX083314). To compare the performance of alu-detect on WGS and exome capture data, we used the sample SRS228129 (50), for which we had both WGS and exome capture data. To compare our results with those of (7), we also ran alu-detect on two data sets used in that study: SRX006833 for NA18506 and SRX016231 for NA18507. We obtained HTS data from the Sequence Read Archive and the European Nucleotide Archive. All data sets analysed consisted of HTS paired 100 bp Illumina reads, except for SRX006833, which consisted of 36 bp Illumina paired reads. We also analysed exome capture HTS data from 96 individuals part of the FORGE Canada project (http://care4rare.ca/).

Detection was run using both NCBI build 36 (hg18) and build 37 (hg19) human reference genomes. We used Alu calls against hg18 to compare with Alu calls made by other studies (all of which use hg18). We used calls against hg19 to compute nick site preferences and in the enrichment analysis. For exome capture data, we initially produced calls against hg18; then we translated them to hg19 using the liftOver tool. We used GENCODE v12 gene annotations (51). We labelled a locus ‘coding’ if for any transcript it is part of coding sequence; ‘non-coding exon’ if it is not part of coding sequence for any transcript, and there is a splicing variant where it is part of an exon, and ‘an intron’ if it is neither coding nor exonic, and it is part of an intron for any transcript.

### Exome capture data

To compare Alu insertion calls made with WGS and exome capture HTS data, we used the SRS228129 sample. WGS calls were filtered as explained earlier, by choosing a filter based on performance when run against the faux reference. To be reasonably sure that those Alu insertions actually intersected (or were close to) the capture kit and exons, we only considered WGS calls with a confidence interval <100 bp (calls with larger confidence intervals are almost always made with no breakpoints detected, and their position can be imprecise.).

We could not use the faux-reference simulation to set parameters for the exome data because there are not enough recently inserted Alus in the proximity of the probes/exons. Instead, we filtered exome capture data with the following parameters: len 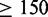; supp 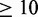; null 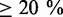; ci-len 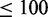 (refer to the Filters subsection for definitions). In particular, every exome capture Alu call was required to be supported by at least 10 read pairs. The exome data sets vary widely in coverage (100× to 283×), making them difficult to filter consistently. We did not try many parameter settings; final selected settings were validated to have the fraction of identified variants that were AluYs (as opposed to AluS/J) ∼90%, the same ratio observed with WGS data.

## RESULTS

In this section, we start by presenting the results of running alu-detect on seven human genomes (including two trios) and 96 exome data sets, comparing our results with those of previous methods. We then use alu-detect to explore the nick-site preferences of Alus insertions and also explore the distribution of novel Alus in the genome while correcting for these preferences.

### WGS data

We ran alu-detect on WGS data from seven individuals, including two trios. The results are summarized in Table 1. We detected on average 1519 novel Alus per individual. The number of Alus is correlated with individual ancestry: there were on average 1339 and 1718 Alu calls per CEU and Yoruban individual, respectively.

**Table 1.** Summary of calls on WGS data sets

Sample	cv	tlen	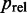	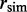	sup	calls	1 brk	2 brk
NA12877	55	322	0.707	0.834	13	1328	1327	346
NA12878	57	322	0.694	0.841	12	1379	1337	329
NA12882	54	322	0.702	0.835	13	1310	1310	367
NA18506	44	310	0.596	0.864	6	1727	1570	429
NA18507	45	311	0.602	0.864	7	1634	1492	419
NA18508	45	302	0.590	0.864	8	1794	1656	511
SRS228129	41	329	0.671	0.865	6	1461	1267	336

We show coverage and mean template length for all HTS data sets (The reads were always 100 bp paired.).

All calls were made by selecting a filter with simulated precision 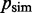 of at least 0.97 and maximizing recall. We display relative precision 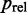 [percentage of calls in (8) or (48)], simulated recall 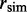, the number of read pairs required to support each call based on our parameter selection simulation (supp), the total number of filtered calls (calls), those with at least 1 breakpoint detected and those with both breakpoints detected.

**Figure 5.**
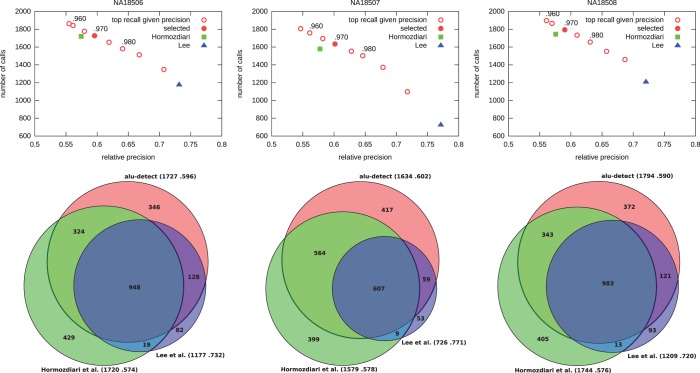
Alu calls on the Yoruban trio. Columns, in order: NA18506 (son), NA18507 (father), NA18508 (mother). Top: Number of calls versus relative precision achieved by alu-detect. For *x* ranging from 0.920 to 0.990 in steps of 0.005, we show the filter parameters that achieve simulated precision at least *x* and maximizes simulated recall (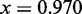 is highlighted). We also show data points corresponding to the results of (6) and (7). Bottom: Intersections of calls on each sample between studies (area-proportional). Next to the study identifier: calls made and relative precision (fraction of the calls also present in dbRIP or Stewart *et al.*). The numbers in the diagram do not always add up because interval intersection is non-transitive.

In Figure 5A, we show the number of calls versus relative precision [fraction of our predictions that are in the data sets of (8) and (48)], which we achieve on the Yoruban trio by varying the target simulated precision from 0.920 to 0.990 in steps of 0.005. We also display data corresponding to (6) and (7).

In Figure 5B, we show the comparison of our Alu calls with those made by (6) and (7) on the Yoruban trio. Compared with (6), we make more calls, but fewer of them are unique to our study, which suggests better precision. Compared with (7), we have higher recall, making on average 769 more calls, of which 515 are previously identified Alus by other studies, but our relative precision is lower. We emphasize this is a comparison of the results, not of the methods behind the results, because different studies used different HTS data sets for the same individuals.

To directly compare our method with the method of (7), we ran alu-detect on two of the HTS data sets included in that study: SRX006833 (36 bp Illumina reads) for NA18506 and SRX016231 (100 bp Illumina reads) for NA18507. We compared the Alu insertion calls in (7) and those made by alu-detect with two other sets of calls: those in (8) and (48), (mostly calls made in other individuals, and hence more useful for evaluating precision) and calls in (6) for that same individual (and more useful for evaluating recall). The results, summarized in Table 2, demonstrate the complementary strengths of the methods: the method of (7) has higher precision, whereas our method has better recall. The difference in recall is especially noticeable with newer 100 bp reads used for the NA18507 data set, which are more amenable to our split-read mapping approach. On these data, alu-detect has overall nearly double the recall of (7) (0.732 versus 0.390), with 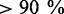 of the calls detected with one or more breakpoint. Overall, alu-detect achieves a higher f-score (harmonic mean of precision and recall) compared with (7).

**Table 2.** Comparison of calls made by (7) and alu-detect on the same HTS data set

set	dbRip + Stewart				Hormozdiari		
	n.calls	prec.	recl.	f-scr.	prec.	recl.	f-scr.
NA18506: 36 bp paired Illumina							
Lee	1177	0.732	0.184	0.293	0.822	0.562	0.668
ad, all	1615	0.628	0.216	0.322	0.765	0.719	0.741
ad, 1 brk	1275	0.653	0.178	0.279	0.780	0.578	0.664
ad, 2 brk	281	0.705	0.042	0.080	0.829	0.135	0.233
NA18507: 100 bp paired Illumina							
Lee	726	0.771	0.119	0.207	0.848	0.390	0.534
ad, all	1646	0.587	0.206	0.305	0.702	0.732	0.717
ad, 1 brk	1543	0.614	0.202	0.304	0.730	0.714	0.722
ad, 2 brk	734	0.666	0.104	0.180	0.767	0.357	0.487

We break down the calls made by alu-detect into all, those made with at least one breakpoint detected, and those made with both breakpoints detected. We show precision, recall and f-score of these call sets compared with two other calls sets: calls in dbRIP (6) and calls in (8) and (48) (mostly in other individuals), and calls made by (6) in that same individual.

### PCR validation of individual variants

We worked with The Centre for Applied Genomics (Toronto) to perform PCR validations on 10 random calls with one breakpoint detected and 10 random calls with two breakpoints detected. PCR primers were designed to be ∼100—150 bp away from the confidence interval of each call. Thus, the reference allele was expected to generate a product of ∼250—350 bp, whereas an allele with a novel Alu would generate an amplification product of >550 bp.

Of the 10 calls with one breakpoint detected, six (three heterozygous, three homozygous) were validated by the PCR experiment, whereas of the 10 calls with two breakpoints, three PCRs failed, whereas of the seven that generated a product, six validated the insertion (five heterozygous, one homozygous). Interestingly, when we inspected the five calls that only generated the reference allele (and thus appear to be false positives), we found that each of them is also reported (in multiple individuals) by all of (6), (7), and (8). Moreover, two of these five calls also appear in dbRip (48), and one of them was PCR validated by a previous study. Thus, although these calls may be false positives, they may also be poorly accessible to PCR validation, for example, because our probes were placed across the breakpoints of the novel insertion, or the amplification product with the novel insertion is too long for PCR.

We also applied Sanger sequencing to the products of the six calls with two breakpoints detected that showed evidence of an insertion allele. Five of the six Alu head breakpoints and five of the six Alu tail breakpoints were confirmed as called by alu-detect either through automatic or manual inspection of the traces (sequencing quality drops significantly at the breakpoint owing to a mixture of the reference and insertion alleles). The remaining Alu head breakpoint was miscalled by 12 bp owing to the presence of reference Alu at the insertion position, whereas the remaining Alu tail breakpoint was inconclusive (the trace was unreadable).

### Non-mendelian calls

The rate of Alu insertions is estimated to be about 1 in 20 births (52) and (14). Having analysed two trios, we expect to see most likely none, and at most one or two Alu calls in the children that are not present in either parent. For the Yoruban trio, there are 88 calls made in the son (NA18506) that do not match any calls from either parent (NA18507, NA18508). However, after removing all Alu call thresholds (e.g. only requiring two fragments to support the insertion; see ‘Materials and Methods’ section for full list of parameters), only 4 of 88 do not match any unfiltered call from either parent. Moreover, one of these calls appears in (8), and the others were labelled as part of the AluSx subfamily, which is no longer active, making it unlikely they are authentic *de novo* insertions in the child. In the case of the Central European trio, there are 70 calls in the son (NA12882) that do not match calls from either parent (NA12877, NA12878), but only 6 of 70 do not match any unfiltered call in the parents. On closer inspection, the alignment score for each of these calls is low, and some of them overlap existing Alus in the reference, making it more likely they are false positives. In general, the dramatic drop in candidate *de novo* events when we remove filter thresholds in the parents suggests that these non-Mendelian calls are much more likely to be false negatives (in the parents) rather than false positives (in the children), which is consistent with our simulation results.

### Alu detection from exome capture sequencing

An important feature of alu-detect is its ability to identify novel retrotranspositions in the immediate proximity of exons from whole-exome capture sequncing experiments. Exome capture data typically have additional biases compared with WGS, including uneven coverage and strand bias at the ends of the capture probes. To evaluate the accuracy of alu-detect on such data, we compared the Alu insertion calls made with genomic and exomic data for the SRS228129 individual from (50), for which the genomic data set had 41× coverage (SRX083314) and the exomic one was generated with the Agilent SureSelect Human All Exon 50 Mb kit and had 100× coverage (SRX083311).

We identified Alu insertion calls with alu-detect in three (overlapping) sets of regions: all exons (coding and untranslated), coding exons (excluding untranslated) and locations of the probes in the exome capture kit (the probes target most, but not all exons, and often do not fully span them). With these calls serving as the ground truth, we then evaluated the ability of exome capture data to identify the same variants. To emphasize the effect of variability in sequencing coverage, we separately show data for regions where exome capture data had coverage of at least 10×, computed using 100 bp windows. The results are summarized in Table 3. Overall, 9/12 (75%) of genomic calls that took place inside the directly targeted regions could be retrieved with exome capture data, whereas the performance degraded if the insertion location was outside of the probe. This was especially notable in non-coding exons, which typically have fewer probes.

**Table 3.** WGS versus Exome Capture

Region	GS	XS	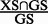	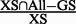
Coding exons	12	10	5/12 = 0.42	8/10 = 0.80
Exons	25	11	6/25 = 0.24	9/11 = 0.82
Probes	12	12	9/12 = 0.75	11/12 = 0.92
Coding exons, coverage 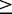 10×	9	10	5/9 = 0.56	8/10 = 0.80
Exons, coverage 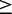 10×	11	11	6/11 = 0.55	9/11 = 0.82
Probes, coverage 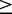 10×	12	12	9/12 = 0.75	11/12 = 0.92

GS: calls from WGS HTS data, with CI 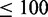.

XS: calls from exome capture HTS data, with CI 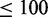. All-GS: calls from WGS data, without the CI filter. These are proximal to the genomic features but may not directly intersect them. XS 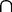 GS/GS: calls made with WGS data in the corresponding region that were also found with exome capture data in that region, i.e. ‘recall’ of exome capture relative to WGS. XS 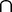 All-GS/XS: calls made with exome capture data in that region that match some call made with WGS data, i.e. ‘precision’ of exome capture relative to WGS. For Exons and Coding Sequence, we include the full set of regions from our annotation, regardless of whether they are captured by the kit. Bottom rows: Genomic calls are restricted to regions where exome capture data have coverage of at least 10×. Coverage was computed by tiling the genome with 100 bp windows. All regions are extended by 50 bp.

To further evaluate the ability of alu-detect to identify novel retrotranspositions from exome capture data, we ran the tool on a data set of exomes generated by the FORGE project, a Canadian consortium for the study of rare disorders. We ran our method on 96 samples from this repository. The mean coverage was 160× (min: 67×, max: 283×). After filtering, we found an average of 7.8 Alu calls per sample (min: 1, max: 20, total: 749). Only 243 calls were within 50 bp of an exon, of which 28 were unique, with the following breakdown: 59 in total (unique: 9) interrupted a coding exon; 30 in total (unique: 4) interrupted a non-coding exon; 86 in total (unique: 8) fell within 50 bp of a 3′ end of an intron, potentially disrupting the splice site or the polypyrimidine tract; and 68 in total (unique: 7) fell within 50 bp of a 5′ end of an intron.

Although most of the disorders within FORGE are solved, a small fraction does not have an identified causative mutation. To determine whether any were due to an Alu, we filtered out the Alu calls occuring in more than five samples between the samples in FORGE, as well as (6), (7), (8), and (48). We were left with five potential Alu insertions investigate: one interrupting the coding sequence of ACAD11, one interrupting an exon downstream of the stop codon of TTLL5, and three potentially interrupting the polypyrimidine tract in introns of LOC646278 (3 bp upstream of a coding exon), FASTKD1 (27 bp upstream of an exon) and GNE (17 bp upstream of an exon). None of these calls segregated between affected and non-affected individuals in the respective pedgrees, indicating they are not causative. However, they demonstrate the types of analyses that can be performed with alu-detect using exome capture data only.

### EN nick site preferences

We used the Alu calls made on the WGS data sets to analyse the distribution of EN nick sites that lead to a successful Alu integration. We applied more stringent filtering criteria than before, keeping only those calls for which we were able to detect both breakpoints, which had an inner length of at least 300 bp, and which had a TSD of length <50 bp (consistent with TPRT). If two or more calls were overlapping between the seven analysed individuals, we kept only one such call. In total, we were left with a set of 961 high-confidence Alu insertion calls.

First, for every 6mer *s*, we compute the EN nick site preference for *s* as:

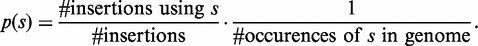



Intuitively, this is the probability that a new insertion cleaves the genome at a specific location, given that *s* is the 6mer at that location. The results are presented in Table 4. We observe that EN prefers TT/AAGA as much as the more widely reported TT/AAAA The absolute count of nick sites of the latter form is larger only because that 6mer has a higher number of occurences.

**Table 4.** Site preferences

6mer *s*	Use(*s*)	Count(*s*)	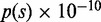
TTAAGA	66	2 288 139	299.8
TTAAAA	187	6 962 385	279.1
TTAGAA	31	2 649 151	121.6
ATAAGA	24	2 079 719	119.9
ATAAAA	64	6 336 702	104.9
GTAAGA	12	1 291 580	96.5
TTGAAA	27	3 520 814	79.7
TTAAAG	20	2 683 435	77.4
GTAAAA	17	2 572 529	68.6
AGAATT	17	3 081 468	57.3
CTAAAA	18	3 495 396	53.5
AGAAAT	21	4 618 715	47.2
TCAAAA	19	4 226 703	46.7
AGAAAA	30	7 090 365	43.9
AGAAAG	15	4 056 864	38.4
ACAAAA	20	5 675 151	36.6
ATGAAA	13	3 884 418	34.7
TTAAAT	11	4 192 065	27.2
TTTAAA	10	6 870 404	15.1

The probability that a new Alu insertion by TPRT will initially cut the genome between positions *x* and *x* + 1, as a function of the 6mer at positions 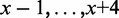. use(*s*): the number of times we observe the kmer *s* used by an Alu insertion. count(*s*): the number of times *s* appears in the (double stranded) genome.

### Alu distribution

As the process of Alu insertions is not completely understood, it is not clear whether it depends on factors other than the genomic 6mer at the initial EN nick site. For example, (28) observes that following an Alu insertion the initial EN cleavage site continues to exist at the 5′ end (‘head’) of the Alu, but not at its 3′ end (‘tail’). However, analysis of 27 (likely fixed) insertions, showed approximately equal numbers of insertions next to the heads and tails of existing Alus (28). We used alu-detect, including 961 polymorphic insertions identified in seven genomes to evaluate whether we can observe any statistically signifcant enrichment or depletion of Alus in proximity to reference Alus.

Having computed EN nick site preferences, we consider the distribution 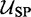 of Alu insertions, where EN nick sites are chosen solely on the basis of the 6mer at that site. Specifically, the probabilty that a new Alu will be inserted on the positive strand between positions *x* and *x* + 1 equals *p*(*s*), where 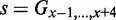 is the 6mer at positions 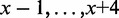 of the genome:
(1)
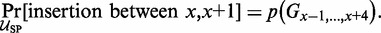



Using Equation (1), the probability under 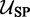 that an Alu will be inserted in a stranded segment *S* is the sum of probabilities that it will be inserted on the positive strand in between every 2 consecutive locations in *S*:

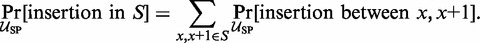



For double-stranded segments, we sum over both positive and negative strands. If *S* is the whole genome, the probability of insertion being in S is 1.

An equivalent way of describing 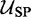 is as follows. For every insertion: (i) a 6mer is chosen using the observed distribution of 6mer nick sites, then (ii) an actual nick site is chosen uniformly at random among all locations in the genome where that 6mer appears. Furthermore, insertions are independent of each other. We refer to 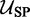 as ‘uniform modulo nick site preferences’. For comparison, we also include tests of how well the completely uniform distribution 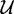, where every Alu is placed at a location chosen uniformly at random, models Alu insertions.

When *n* Alu insertions are observed, the number that are inserted in *S* should follow the binomial distribution 
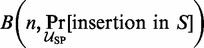
. We thus test for statistically significant enrichment or depletion of Alu insertions under 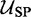 and 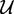 in various segments *S* using a single-sided binomial test. The set of regions tested included exonic regions as well as regions in the vicinity of existing Alus (see ‘Materials and Methods’ section for boundary definitions). The results are presented in Table 5.

**Table 5.** Alu insertions in the exome and in other Alus

Region	Obs	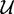		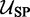	
		exp	*P*-value	exp	*P*-value
Coding sequence	0	11.0	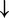 0.**000**	5.4	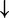 0.**011**
Exons (untranslated)	9	21.8	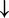 0.**004**	19.6	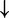 0.**011**
Introns	472	463.3	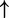 0.297	499.0	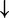 0.**044**
Inner introns	469	458.6	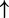 0.262	493.9	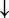 0.057
Genes	479	496.0	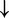 0.144	524.0	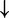 0.**002**
Upstream 200bp	1	2.1	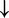 0.302	1.4	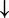 0.499
Alu head region (+)	6	10.3	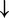 0.117	13.5	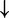 0.**028**
Alu head region (−)	9	10.3	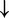 0.403	8.9	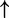 0.557
Alu middle region (+)	10	13.9	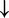 0.177	10.0	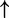 0.559
Alu middle region (−)	1	13.9	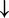 0.**000**	1.6	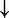 0.427
Alu tail region (+)	20	10.3	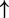 0.**002**	17.2	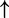 0.288
Alu tail region (−)	8	10.3	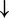 0.287	8.4	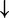 0.519
Alu overall (+)	36	34.5	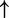 0.430	40.7	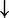 0.250
Alu overall (−)	18	34.5	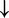 0.**003**	18.9	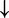 0.465

There were 961 trials in total. For exome-related regions, we consider insertions in either strand together. For Alu-related regions, we consider insertions in the same (+) and opposite (–) strands separately. For precise region definitions see ‘Materials and Methods’ section. 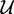: Alu insertions are completely uniform over the genome. 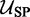: Alu insertions are uniform modulo EN nick site preferences. Up/Down arrows: observation is larger/smaller than expectation.

The results for genic regions are as expected: we observe a statistically significant depletion of Alu insertions in both exons and introns, and no insertions at all in coding sequence, likely due to functional constraint. More interestingly, once we correct for nick site preferences, we observe no statistically significant enrichment of Alu insertions in various regions around reference Alus. This suggests that EN nick sites that lead to successful Alu integration are not influenced by factors that would continue to exist in the genome following an Alu insertion, for otherwise we would have observed an enrichment of Alu insertions in the vicinity of other Alus.

Although there appears to be a slight depletion of Alu insertions on the positive strand in the head region of reference Alus (*P* 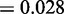), we believe this is an artefact of the analysis methodology. The alu-detect is more ‘reluctant’ to call Alu insertions in on the positive strand of reference Alus (see the ‘Filtering’ section under ‘Materials and Methods’ section). This likely results in lower recall in those regions. To evaluate its extent, we computed the simulated precision and recall (using the faux-reference simulation) of our method both overall, and for Alu insertions on the positive strand near reference Alus. The ‘high-confidence’ calls used in the enrichment analysis achieve 0.990 simulated precision and 0.547 simulated recall throughout the genome, and 0.961 simulated precision and .470 simulated recall on the positive strand near ‘clear’ Alus. Precision and recall for Alu insertions on the negative strand of existing Alus is similar to the genome overall. (The recall numbers are lower than those shown earlier, e.g. Table 1, as here we require both breakpoints to be detected for the call to be made.) Thus, recall for insertions on the positive strand near ‘clear’ Alus is ∼15% lower than recall in the genome as a whole. If, to correct for this, we add 15% to the number of observed calls in the rows of Table 5 corresponding to regions near ‘clear’ Alus on the positive strand, the depletion observed near the head of reference Alus is no longer significant (*P* 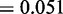).

## DISCUSSION

### Nick site preferences

The breakpoint detection ability of alu-detect enabled us to study the ‘preferences’ of EN nick sites that lead to successful Alu integration using novel Alu insertion calls from seven samples. Nick site ‘usage’ was analysed by (29) (linearly related to preference), but using Alus insertions obtained by comparing the NCBI and Celera genomes. Our results are overall similar, but there are differences. In this study, we find that the top two preferred sites are TT/AAAA and TT/AAGA. They are comparable with each other, and the preference for any other site is smaller by a factor of two. In contrast, (29) find that GT/AAGA and AT/AAGA have larger preferences. This discrepancy may be explained by the fact that among all relevant 6mers, these two have the lowest absolute counts in the genome (see Table 4), therefore possibly the largest variance of observed site preference between individuals.

### Parameter selection

Our proposed faux-reference simulation works to eliminate two biases: First, each Alu ‘target’ that we remove from the real reference (creating a ‘fake’ reference) was inserted at some point during evolution. Second, we do not simulate HTS data, instead, using real HTS data to detect the Alu targets.

For alu-detect, the simulation was an integral part of the runs on WGS data, as it is used to set filtering parameters for Alu insertion calls. The central assumption in this process is that precision and recall achieved in detecting Alu targets against the faux reference is closely related to precision in detecting novel Alus against the real reference. Our results show that this assumption holds for our data sets, and our method for setting parameters should generalize to other data.

Even though two common major biases were eliminated, some biases remain. First, during the simulation, an Alu detected on the faux reference that matches a known novel Alu is removed from consideration. In doing that, we are assuming they are more likely real novel Alus in the donor. This can introduce a bias because if the Alu is not present in the donor, the call should be considered a false positive of the simulation. This bias should be small because the fraction of locations where false positives can be missed in this way is small. Second, true novel Alus in the donor that are not previously known might appear as false positives of the simulation, leading to slight under-estimate of precision, though the effect should be small. Finally, young Alus have longer and cleaner poly-A tails, and more generally, reference Alu targets are close enough to their respective Alu consensus sequence to permit annotation, whereas true novel Alus do not have this restriction.

### Future directions

One notable feature not currently implemented in alu-detect is the ability to differentiate between homozygous and heterozygous calls. Conceptually, to make this distinction, one must re-inspect the reference mappings after producing Alu insertion calls to look for evidence supporting the reference allele at the locations of those calls.

Furthermore, alu-detect enables other analyses. One possible direction would be to locate, for each Alu insertion, its potential origin (the reference Alu that it was copy-pasted from), in the hope of identifying active Alu elements.

## CONCLUSION

The alu-detect software tool detects novel Alu insertions directly from HTS reads, with flexible tradeoffs between precision and recall. Although all data sets analysed consisted of (state-of-the-art) 100 bp paired reads, the tool itself is designed to work with other types of base-space HTS data: paired or unpaired, and shorter or longer in length. Our method improves on previous work in several ways:

### Identifying Alu breakpoints and TSDs

The alu-detect attempts to identify the precise breakpoints of each Alu insertion and identify the full length of the TSD; a confidence range is only reported when the breakpoints could not be confidently mapped.

### Identifying Alus from exome capture HTS

The exome capture kit affects the distribution of reads across the sequenced regions. To our knowledge, alu-detect is the first method for novel Alu identification that works with exome data.

### Alus in the vicinity of other Alus

The alu-detect can identify Alu insertions in immediate proximity of other Alus, whereas most previous methods make no calls in such regions.

### Distribution of Alus across the genome

We use observed nick site preferences to identify areas of increased or reduced Alu activity across genes and other Alus in the reference. We demonstrate that although there appear to be statistically significant enrichments and depletions of Alus in regions proximal to other Alus, this can be explained by nick site preferences, and thus the DNA content of Alus. This argues against Alu insertions being affected by a factor that would continue to exist in the genome following an Alu insertion, e.g. an upstream (or downstream) motif that remains after an Alu insertion.

## DATA ACCESS

The alu-detect is available at http://compbio.cs.toronto.edu/alu-detect/. Access to the FORGE repository is available through http://care4rare.org/.

## SUPPLEMENTARY DATA

Supplementary Data are available at NAR Online.

## FUNDING

Canadian Institutes for Health Research (CIHR) Catalyst Grant and Ontario Research Fund (ORF) GL2 grant (to M.B.). Funding for open access charge: CIHR and Sloan Foundation.

*Conflict of interest statement*. None declared.

## Supplementary Material

Supplementary Data
