# Supplementary material for: Detecting Alu insertions from high-throughput sequencing data
Source: Nucleic Acids Res. 2013 Aug 5;41(17):e169. doi: 10.1093/nar/gkt612 (PMC3783187; doi:10.1093/nar/gkt612)
Supplement: Supplementary Data [file supp_41_17_e169__index.html]

Detecting Alu insertions from high-throughput sequencing data — Detecting Alu insertions from high-throughput sequencing data — Supplementary Data 

# Detecting Alu insertions from high-throughput sequencing data

## 

files

**Files in this Data Supplement:**

- Supplementary Data - xls file
- Supplementary Data - doc file
- Supplementary Data - doc file
