# Supplementary material for: Detecting Alu insertions from high-throughput sequencing data
Source: Nucleic Acids Res. 2013 Aug 5;41(17):e169. doi: 10.1093/nar/gkt612 (PMC3783187; doi:10.1093/nar/gkt612)
Supplement: Supplementary Data [file supp_gkt612_nar-00183-met-k-2013-File002.doc]

**Alu insertions identified with 2 breakpoints**


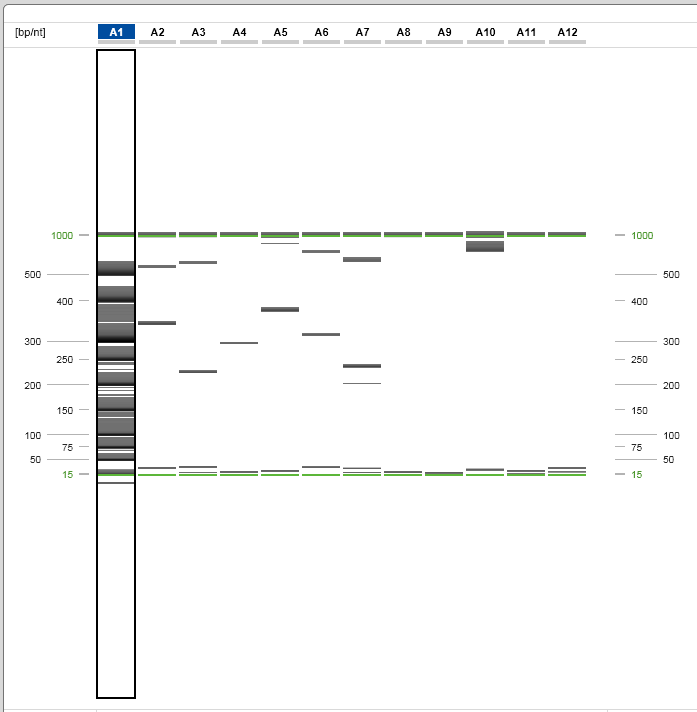


| 2 - "random 2bp" group | | | |  |  |
| --- | --- | --- | --- | --- | --- |
| Order | Chr | Number | Well |  |  |
| 1 | 2 | 65110447 | A2 | 316 bp | |
| 2 | 14 | 36960786 | A3 | 202 bp | |
| 3 | 1 | 179124171 | A4 | 261 bp | |
| 4 | 13 | 89348560 | A5 | 344 bp | |
| 5 | 4 | 171071165 | A6 | 274 bp | |
| 6 | 9 | 38757759 | A7 | 209 bp | |
| 7 | 7 | 96905456 | A8 | 298 bp | |
| 8 | 10 | 55851225 | A9 | 312 bp | |
| 9 | 18 | 25410352 | A10 | 338 bp | |
| 10 | 20 | 12491587 | A11 | 179 bp | |
|  |  |  |  |  |  |
| A1 - DNA ladder | | |  |  |  |
| A12 - NTC (used primer pairs 1 & 10) | | | | |  |
