# Supplementary material for: Detecting Alu insertions from high-throughput sequencing data
Source: Nucleic Acids Res. 2013 Aug 5;41(17):e169. doi: 10.1093/nar/gkt612 (PMC3783187; doi:10.1093/nar/gkt612)
Supplement: Supplementary Data [file supp_gkt612_nar-00183-met-k-2013-File003.doc]

**ALU Insertions identified with 1 breakpoint**


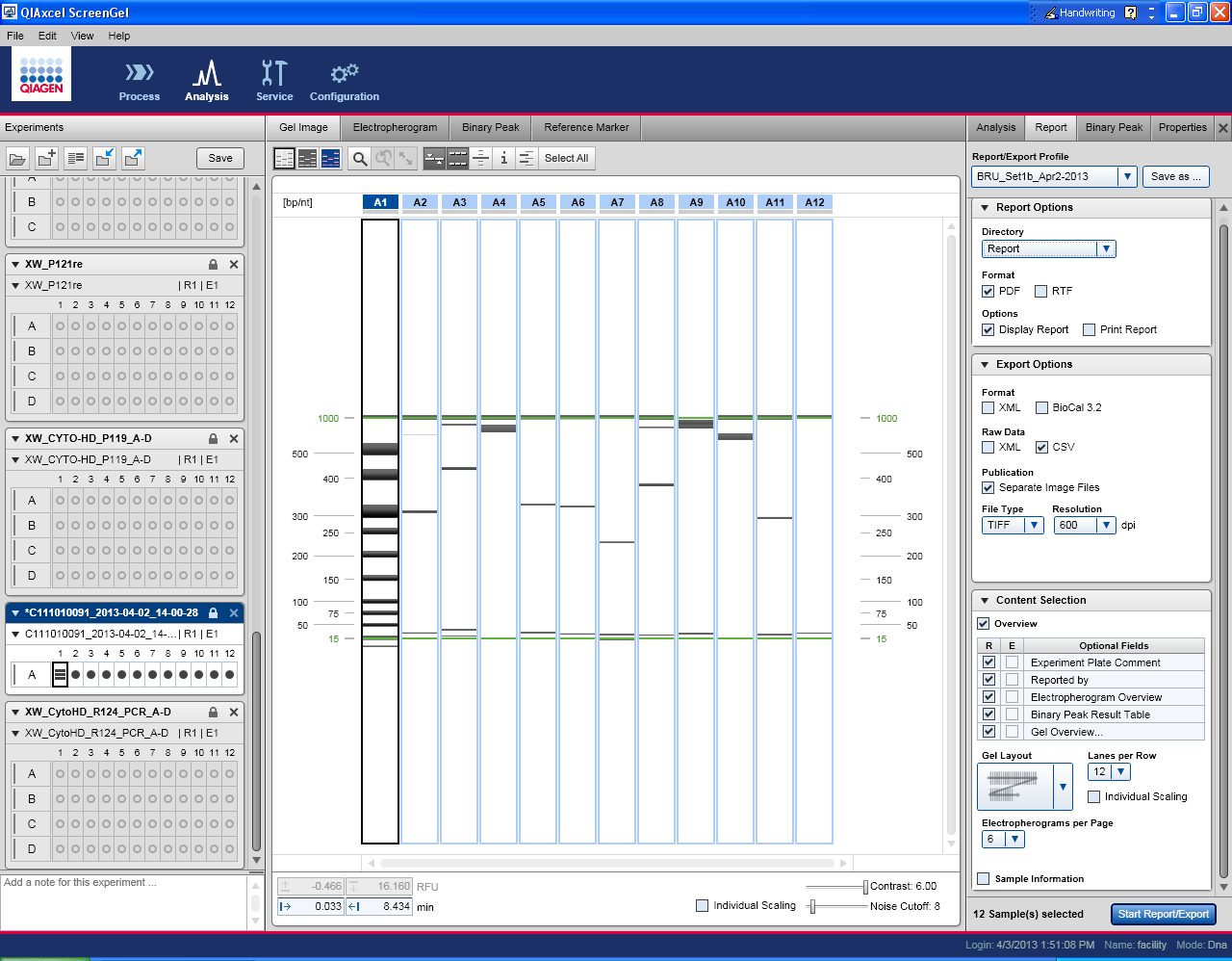


| | 1 - "random 1bp" set | | |  | | --- | --- | --- | --- | | Order | Chr | Number | Well | | 1 | 1 | 111802743 | A2 | | 2 | 8 | 130024898 | A3 | | 3 | 13 | 31133620 | A4 | | 4 | 4 | 68627323 | A5 | | 5 | 12 | 27403170 | A6 | | 6 | 7 | 6280571 | A7 | | 7 | 2 | 178252060 | A8 | | 8 | 20 | 52071305 | A9 | | 9 | 3 | 43567167 | A10 | | 10 | 11 | 102030679 | A11 |   A1 – DNA ladder  A12 – NTC (used primer pairs 1 & 10) |  |  |
| --- | --- | --- | --- | --- | --- | --- | --- | --- | --- | --- | --- | --- | --- | --- | --- | --- | --- | --- | --- | --- | --- | --- | --- | --- | --- | --- | --- | --- | --- | --- | --- | --- | --- | --- | --- | --- | --- | --- | --- | --- | --- | --- | --- | --- | --- | --- | --- | --- | --- | --- |
